# Bone Health Nutrition Issues in Aging

**DOI:** 10.3390/nu2111086

**Published:** 2010-11-08

**Authors:** Karen Plawecki, Karen Chapman-Novakofski

**Affiliations:** Division of Nutritional Sciences, University of Illinois at Urbana-Champaign, Urbana, IL 61801, USA; Email:kmc@illinois.edu

**Keywords:** calcium, vitamin D, bone, aging

## Abstract

Bone health is an important issue in aging. Calcium and vitamin D currently have the most focus in published research on nutrition and bone health in aging, although evidence from published research is not conclusive. A systematic review was conducted to determine the impact of dietary and supplemental interventions focused on calcium and vitamin D over the past 10 years. Using key words to search, and search limits (aging population, English), 62 papers were found related to diet, nutrition, and bone; and 157 were found related to calcium and bone. Our review found a positive effect on bone health for supplements; food-based interventions; and educational strategies. Although there may be a publishing bias related to non-significant findings not being published, our results suggest the effectiveness of food based and educational interventions with less economic impact to the individual, as well as less risk of physiological side effects occurring.

## 1. Introduction

Osteoporosis is a systemic skeletal disease portrayed by low bone mass and structural weakening of the bone material that leads to reduced bone strength and increased susceptibility to fracture. Although all bones can be affected, the hip, vertebra and wrist bones are at high risk. Osteoporosis is commonly referred to as a “silent disease” as there are no symptoms until the fracture occurs [1].

Osteoporosis is a debilitating chronic disease that is a public health problem. It is estimated that 10 million individuals have osteoporosis while another 34 million suffer from low bone density. An estimated 61 million individuals will develop osteoporosis or low bone density by 2020 [1]. In 2002 annual direct care expenditures for osteoporotic fractures reached almost 18 billion dollars [2,3]. Beyond costs, there is the physical burden of living with osteoporosis and its impact on daily living, including restrictions in daily activities, loss of confidence (due to fear of falling and fracture) and loss of independence [4].

With people living longer, fracture risk is expected to increase. Each year an estimated 1.5 million individuals suffer a fracture due to bone disease. For those over 50 who suffer a hip fracture, approximately 4% will die in the hospital and 24% will die within the year. The risk of a fracture increases with age and is greatest in women. Approximately one in two women and one in four men aged 50 or older in the United States will experience an osteoporotic-related fracture sometime during the remainder of their lives. As people live longer, the lifetime risk of fractures will increase for all ethnic groups [2]. With the high costs of treating osteoporosis fractures [5], effective preventive interventions are needed.

Osteoporosis is primarily viewed as a woman’s disease. However, after age 50, 6% of all men will experience a hip fracture and 5% will have a vertebral fracture as a result of osteoporosis [1,2]. Bone loss occurs rapidly in women at menopause; for men the loss still occurs but later (in their late 60s and in their 70s). The topic of bone health and osteoporosis in men is not well studied, but it is believed to often occur due to secondary causes, such as corticosteroid therapy [6].

Risk factors for low bone density, osteoporosis and fractures, include both unchangeable and modifiable types of factors. Conditions increasing the chance for developing osteoporosis include: fracture history after age 50, family history, female gender, small bone frame, advanced age, estrogen deficiency, amenorrhea, low testosterone levels, some medications, certain chronic diseases, long-term low intake of calcium, vitamin D deficiency, inactivity, cigarette smoking and excessive alcohol [7]. Fortunately, there are many modifiable lifestyle factors (diet and activity) along with drug treatment which can prevent or slow the loss of bone.

The National Osteoporosis Foundation developed five steps to optimize bone health [1]. These include:

1. Get the daily recommended amounts of calcium and vitamin D;
2. Engage in regular weight-bearing and muscle-strengthening exercise;
3. Avoid smoking and excessive alcohol;
4. Talk to your healthcare provider about bone health;
5. Have a bone density test and take medication when appropriate.

Clinical studies have reported increases in calcium intake as a result of calcium supplementation and physical activity and intense supervision [2,8,9]. There is an inverse relationship between physical activity and future hip fracture risk for both women and men [10,11]. Resistance exercise combined with aerobic weight-bearing activity has been shown to improve bone mineral density (BMD) in postmenopausal women without a history of fractures. Calcium and vitamin D supplementation have been shown to increase BMD, but even within clinical trials adherence to taking supplements is not optimal. In fact, one large trial found no change in BMD in women enrolled in the supplement arm of the trial. However, when only those who actually took the supplements on most days were analyzed a significant improvement in hip BMD was found [12]. Clearly, exercise and diet can have a positive impact on bone health.

## 2. Review Section

### 2.1. Objectives

The objectives of this systematic review were to: Assess the impact of dietary intervention on dietary behavior measures, laboratory indices of bone health, bone mineral density, and other variables related to bone health; discuss the results of this review in terms of healthy aging; and identify gaps in the research related to bone health and aging.

### 2.2. Methods

**Figure 1:**
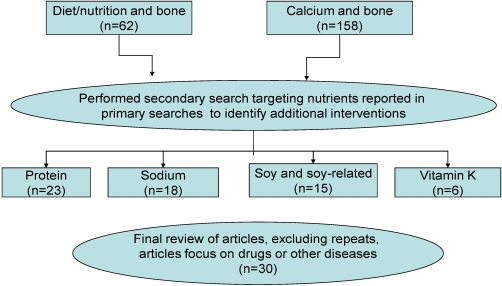
Description of methods for systematic search.

The last 10 years in PubMed were searched using the keywords intervention, diet, and bone, as well as calcium, intervention, and bone (see Figure 1). Studies with vitamin D were included in both primary searches. The search limits included searching only for the classifications of middle-age and aged, humans, and publications in English. A secondary search was conducted on specific nutrients (protein, sodium, soy and vitamin K) which were in the results from the initial searches. The secondary searches were performed to identify additional studies that may not have been included in the initial search. Interventions that included drugs were not included in the review. For the purpose of this review, manuscripts of cross-sectional studies were eliminated.

Data extracted included type of intervention (randomized or quasi-experimental), number of subjects, length of study, description of the intervention, nutrient of interest, and outcome measures. Authors reviewed the manuscripts for consensus in data extraction and interpretation of results.

## 3. Results

These research studies could be placed into one of four groups: calcium and vitamin D from food (Table 1), calcium and vitamin D from supplements (Table 2), other bone health-related nutrients (Table 3) and portfolio diets (Table 4).

**Table 1:** Calcium and vitamin D interventions (Food).

Authors	Date	N	Length	Intervention	Outcome
Moschonis *et al.* [13]	2010	66	30 months	Postmenopausal woman from 55–65 years old were randomized to either diet intervention with counseling or control (usual diet and no counseling). The diet group consumed fortified dairy, 3 daily servings of milk and yogurt for 12 months (1200 mg and 7.5 µg vitamin D/day). After 12 months and for additional 18 months, the vitamin D fortified levels increased to 22.5 µg vitamin D/day.	The intervention group had a significant increase in BMD ^a^ for arms, total spine, and total body *vs.* the control group.
Manios *et al.* [14]	2007	101	12 months	Post-menopausal women, ages 55–65, received either calcium and vitamin D fortified dairy (1200 mg calcium/d and 7.5 µg vitamin D3/d, n = 39), calcium supplementation (600 mg to reach 1200 total Ca/day, n = 36) *vs.* control (n = 36). The dairy group received biweekly education sessions whereby dairy samples were provided at the end of each session. BMD changes were measured by DEXA ^b^ and QUS ^c^. The following bone indices were measured: insulin‑like growth factor (IGF)-I, parathyroid hormone (PTH), osteocalcin and type I collagen cross-linked *C*‑telopeptide levels.	The dairy group showed better changes at the hip site, total spine and total BMD, based on DEXA readings Calcium supplementation or control groups did not show changes.
					The dairy intervention group showed an increase in insulin-like growth factor (IGF)‑I *vs.* supplemental and control groups. Parathyroid hormone (PTH) levels were borderline statistically increased in the control group No significant differences with osteocalcin and type I collagen cross‑linked *C*‑telopeptide levels.
Bonjour *et al.* [15]	2009	37	1 month	Elderly women (mean age 84.8 ± 8.1 years) living in long term care facility consumed calcium and vitamin D fortified soft plain cheese (302 mg calcium, 2.5 µg vitamin D, 14.2 g protein). The bone resorption marker carboxy terminal cross‑linked teleopeptide was targeted but other serum markers were measured.	PTH ^d^ levels were reduced. Levels of insulin-like growth factor-1, osteocalcin and amino-terminal propeptide of type1 procollagen increased.
Hien *et al.*	2009	140	18 months	Post-menopausal women with low calcium intake; nutrition education to improve calcium intake. Control group stayed with usual diet. Calcium intake, PTH and BMD were measured.	The intervention group showed an increase in calcium intake and a decrease in PTH levels.
[16]					
Solomon *et al.* [17]	2006	31,715	3 separate mailings	Elderly participants received education materials in the mail geared towards strengthening knowledge of osteoporosis, calcium and vitamin D intake along with other bone healthy behaviors. The controls did not receive the informational packets.	No change or difference in knowledge, perceived susceptibility or diet was reported between the intervention or control.
Sedlak *et al.* [18]	2005	124	6 months	Postmenopausal women were given specific instruction by health care professionals on bone healthy behaviors, including calcium intake. The control group did not receive the focused instruction.	Calcium intake increased in both groups.
Wong *et al.*	2004	189	4 months	All participants had an osteoporotic fracture. The intervention group received three tailored counseling sessions and the control group received standard care. Dietary intake (calcium, protein and calories) were evaluated.	Calcium intake was increased following the intervention. No change in protein or calories.
[19]					
Heaney *et al.* [20]	1999	204	12 week following 4 week baseline	Men and women, ages 55–85, who commonly consumed below dairy recommendations were given instruction how to meet dairy group recommendation of 3 servings/day.	Following instruction, mean calcium and vitamin D intake improved by 729 mg and 5.8 µg/day, respectively. Other bone nutrients intake (Mg, P, protein) also improved. PTH levels decreased and the bone resorption marker, *N*‑telopeptide excretion, also decreased.

^a^ Bone mineral density;

^b^ Dual energy X-ray absorptometry;

^c^ Quantitative ultrasound;

^d^ Parathyroid hormone.

**Table 2:** Calcium and vitamin D interventions (Supplements).

Authors	Date	N	Length	Intervention	Outcome
Seamans *et al.* [21]	2010	204	22 weeks	Adults (ages ≥64 years) received cholecalciferol supplementation (0, 5, 10, and 15 µg cholecalciferol/d) during winter months on indices of vitamin D status and bone turnover. Mean calcium intake was 874 mg/day.	Increase in serum 25(OH)D ^a^ based on supplemental dosage up to 10 µg/day and decrease in parathyroid hormone *vs.* control. No other serum bone markers affected.
Kuwabara *et al.* [22]	2009	62	30 days	Adults living in long term care facilities received either 200 mg of calcium with or without 800 IU vitamin D.	Serum 25(OH)D levels increased in the supplemented group but the mean level still fell below 20 mg/mL. In those with good compliance, PTH levels were lower in the vitamin D group *vs.* calcium-only.
Hitz *et al.* [23]	2007	11 (hip fractures, control n = 18)	1 year	Patients with either hip or upper extremity fractures received either 1200 mg calcium and 1400 IU cholecalciferol or placebo (including 200 IU cholecalciferol in multivitamin). Lumbar and pelvis BMD ^b^ measured along with biomarkers.	Lumbar BMD was increased in the intervention group.
		23 (upper extremity fractures, control n = 27)			PTH was reduced in those with hip fracture and receiving intervention. Effect of intervention was more effective in those <70 years.
Prince *et al.* [24]	2006	1460	5 years	Elderly women were randomized to take a placebo or 600 mg calcium twice per day. BMD and adverse effects were monitored.	Calcium supplemented group did not reduce fracture risk but if focus on those complying (>80% taking the supplement) then those showed improved BMD and bone strength and reduced fracture incidence *vs.* those taking placebos.
Jackson *et al.* [25]	2006	36,282	7 years	Post-menopausal women (ages 50–79) were assigned to either 1000 mg calcium with 400 IU vitamin D3/day or control group. Fracture rate and bone density were monitored.	Intervention group had slight higher hip BMD. No significant reduction in fracture risk. Kidney stone risk was more elevated in the treatment group.
Di Daniele *et al.* [26]	2004	120	30 months	Peri- and post-menopausal women received calcium and vitamin D supplement. BMD and BMC ^c^ were measured.	The placebo group lost a total of BMD *vs.* the treatment group.
Meier *et al.* [27]	2004	55	2 year	Healthy adults were given vitamin D3 (500 IU) and calcium (500 mg) after a year of observation during the winter season *vs.* the control. Changes in calciotropic hormones and bone turnover markers were tracked.	During the intervention, effects of winter on hormones and bone turnover markers were reversed or negated in the supplemented group.
Sanders *et al.* [28]	2010	2256	3–5 years	Older, free living women (≥70 years) in the autumn season were given a single dose of 500,000 IU vitamin D/year to determine if this would reduce risk for falls and fractures.	The intervention group had higher falls and relative risk for falls than the control group.
Kärkkäinen *et al.* [29]	2010	3139	3 years	Ambulatory women were studied. The treatment group received 800 IU vitamin D with 1000 mg calcium/day *vs.* the control which was given no supplement or a placebo. Number and frequency of falls were monitored.	There was no difference in the number of single or multiple falls between groups at the population level. For the subgroup, the supplemented group reduced the number of multiple falls.
Kärkkäinen *et al.* [29]	2010	593	3 years	Ambulatory women were studied. The treatment group received 800 IU vitamin D with 1000 mg calcium/day *vs.* the control which was given no supplement or a placebo. BMD measurements were studied.	The supplemented group increased total BMD. For those that were deemed compliant (≥80%) in taking supplements, the improvement in BMD (total and femoral) were significant.
Pfeifer *et al.* [30]	2009	242	20 months	Elderly in the community received either 1000 mg calcium or 1000 mg with 800 IU vitamin D. Number of falls and muscle strength were tracked.	Calcium with vitamin D group had reduced quantity of first falls and improvements in muscle strength *vs.* calcium supplemented group.
Flicker *et al.* [31]	2005	625	2 years	Seniors living in residential settings with vitamin D levels at least 25 nmol/L were given vitamin D supplements (10,000 weekly dose and then 1,000 IU/day) or assigned a placebo. Calcium (600 mg) was provided to both groups. Falls and fractures were prospectively measured.	The vitamin D supplemented group had a reduced incident rate for falls and fracturing.

^a^ 25-hydroxyvitamin D;

^b^ Bone mineral density;

^c^ Bone mineral content.

**Table 3:** Interventions using bone-healthy nutrients addition to calcium and vitamin D.

Authors	Date	N	Length	Intervention	Outcome
Protein					
Rapuri *et al.* [32]	2003	489	3 years	Protein as a percentage of calories and effect of calcium intake on bone loss in women (ages 65–77 years) were studied in this cross‑sectional study. BMD was tracked.	Highest protein quartile (mean 78 g) was associated with higher spine, radius and total BMD ^a^ for those with higher calcium intakes (over 400 mg) *vs.* lower protein intake. No change in hip BMD.
Sodium					
Teucher *et al.* [33]	2008	11	20 weeks (with 5 phases and 4 week washout periods)	Crossover study (5 weeks each phase) with post-menopausal women consumed calcium (low = 518 mg and high = 1284 mg calcium) and salt (3.9 g = low and 11.2 g = high) diets. Calcium absorption and excretion and biomarkers were measured.	Diet high in salt resulted in increased urinary calcium losses and impacted calcium balance on the high calcium diet. Calcium balance on the low calcium was negative for both low and high salt diets. Calcium absorption was more efficient with low calcium but unaffected by salt intake.
Soy					
Alekel *et al.* [34]	2010	432	36 months	Healthy postmenopausal women with no osteoporosis were given either placebo or calcium and vitamin D with isoflavones (80 or 120 mg). BMD was measured.	BMD declined in all groups. The group with 120 mg of isoflavones was more protective at the femoral neck BMD *vs.* the placebo group.
Wong *et al.* [35]	2009	403	24 months	Postmenopausal women were given calcium and vitamin D supplements with additional soy isoflavone (soy hypocotyl aglycone) of either 80 mg or 120 mg soy isoflavone. BMD and BMC ^b^ changes were tracked.	Participants in the 120 mg soy isoflavone group had smaller loss in total BMD than the placebo.
Cheong *et al.* [36]	2007	13	50 day intervention per phase	Postmenopausal women consumed 43 g soy protein via baked goods and beverages. Each intervention contained soy protein with either 0, 97.5, or 135.5 mg total isoflavones. Order of dosage was randomized.	No change in bone resorption markers (urinary cross-linked N teleopeptides of type I collagen and serum osteocalcin) related to change in dosage.
		(3 way crossover)			
Vitamin K					
Booth *et al.* [37]	2008	452	3 years	Men and women (ages 60–80) received either a multivitamin with either 500 µg/d or no phylloquinone. Both groups received calcium (600 mg elemental calcium/day) and vitamin D (400 IU/day) in a separate supplement. Femoral neck, lumbar and total BMD were measured.	No difference in BMD. Percent of undercarboxylated osteocalcin was lower in the supplemented group, indicating improved vitamin K status.
Bolton-Smith *et al.* [38]	2007	244	2 years	Women (ages ≤60 years) with no osteoporosis were treated with either placebo, 200 µg vitamin K, 400 IU vitamin D and 1000 mg calcium, or vitamin K with vitamin D and calcium. DEXA ^c^ measured wrist and hip bone mineral content.	Women taking the combination of vitamin K, vitamin D and calcium showed an increase in BMC and BMD at the distal radius *vs*. other groups. Those receiving vitamin K had reduced percent of undercarboxylated osteocalcin. Those receiving vitamin D showed increased levels of serum 25(OH)D ^d^ and lower levels of PTH.
Braam *et al.* [39]	2003	181	3 years	Post-menopausal women (ages 50–60) were assigned either a combination of calcium/magnesium/zinc/vitamin D with vitamin K (1 mg vitamin K/day) or combination without vitamin K or placebo. BMD of femoral neck and lumbar area were targeted.	The vitamin K supplemented group had less femoral neck bone loss.

^a^ Bone mineral density;

^b^ Bone mineral content;

^c^ Dual energy X-ray absorptometry;

^d^ 25-hydroxyvitamin D.

**Table 4:** Portfolio diets on bone health.

Authors	Date	N	Length	Intervention	Outcome
Dash Diet					
Lin *et al.* [40]	2007	810	18 months	Men and women (mean age: 50 ± 8.9 years) either followed established guidelines or the DASH diet. Nutrient intakes including calcium, potassium and magnesium were evaluated.	Those following the DASH increased calcium, potassium and magnesium intake *vs.* established guidelines and control groups.
Mediterranean Diet					
Bulló *et al.* [41]	2009	238	12 months	Elderly men and women (ages 60–80 years) were randomized to either control (low fat diet), Mediterranean diet with olive oil supplement or Mediterranean diet with mixed nuts. Changes in bone biomarkers were evaluated. Bone mass was measured by QUS ^a^.	The group consuming mixed nuts had a high acid load. This group had higher PTH ^b^ levels *versus* the other groups but no other biomarkers were significantly different.

^a^ Quantitative ultrasound;

^b^ Parathyroid hormone.

### 3.1. Calcium and Vitamin D Interventions Using Food

Interventions, including food rich in either calcium and/or vitamin D, have shown results with increased calcium intake and improving serum bone markers. Food sources used in the studies were dairy-based, either milk or yogurt [13,14,15]. Food was either provided to meet calcium and vitamin D needs [13,14] or as one source [15]. Moschonis *et al.* [13] studied the impact of fortified dairy (two servings of milk and one yogurt) over 30 months with biweekly counseling on bone health in post‑menopausal women. During the first 12 months, the fortified dairy products provided a total of 1200 mg calcium and 7.5 µg vitamin D. For the final 18 months, vitamin D fortified levels were increased to 22.5 µg/day. The women consuming the fortified foods and receiving education showed an increase in arm, spinal and total BMD, in contrast to those staying on their usual diet and receiving no counseling. In the study by Manios *et al.* [14], participants consumed calcium and vitamin D fortified dairy (provided via three servings of either milk or yogurt) to meet nutrient recommendations *versus* those consuming calcium supplements to reach 1200 mg calcium, or the control received neither “dairy” food nor supplements. Those in the dairy group also received instruction on osteoporosis and bone-healthy behaviors. In the study by Bonjour *et al.* [15], elderly women consumed two servings of vitamin D and calcium-enhanced soft cheese that provided a total of 2.5 µg vitamin D3, 302 mg calcium; 233 mg phosphorus, and 14.2 g protein. Participants tolerated the product and showed a modest increase in bone formation markers (insulin-like growth factor-1, osteocalcin and amino-terminal propeptide of type 1 procollagen) along with a decrease in parathyroid hormone (PTH) from baseline. Consumption of calcium and vitamin D rich foods generally resulted in reduced PTH levels.

In addition to providing food, providing instruction has seen a range of results in improving calcium intake [9,14,16,17,18,19,20]. Hein *et al.* [16] demonstrated the effectiveness of a training course. Participants in the intervention over a period of 18 months learned about osteoporosis, calcium-rich foods, local sources of calcium-rich foods, sample menus reviewed and various skills, including food preparation, were practiced. Those receiving the intervention increased their calcium intake *versus* the control group. The PTH levels were also decreased in the treatment group. Similar topics were addressed in the study by Manios *et al.* [14] where the intervention included fortified dairy servings. The bone density measures were improved in the intervention group *versus* the calcium supplement or control groups. Tailored and individualized education [19,20] yielded improved calcium intake over those receiving materials in the mail [17]. Tussing and Chapman-Novakofski [9] also reported increased calcium intake after an eight-week educational intervention.

### 3.2. Calcium and Vitamin D Interventions Using Supplements

Various calcium and vitamin D dosages have been used to improve bone health markers [21,22,23,24,25,26,27] or reduce falls [28,29,30,31].

A study by Kuwabara *et al.* [22] provided adults in residential facilities with either calcium, with or without vitamin D (800 IU). Even with additional vitamin D, participants did not reach recommended serum vitamin D (25(OH)D) levels but did show reduced PTH levels. In those who had experienced a fracture, supplemental calcium (1200 mg) and vitamin D (1400 IU) resulted in increased lumbar bone mineral density (BMD) and lower PTH; the effect of the supplement was more effective in participants under 70 years old [23]. Women who took a calcium supplement (600 mg twice per day) did not show a reduction of fracture risk for the entire intervention group but those that took the supplement over 80% of the time did have improved bone markers [24]. Another study evaluating calcium (1000 mg) and vitamin D (400 IU) in post-menopausal women demonstrated that the treatment group had a slightly higher hip BMD but no lower fracture risk [25]. Calcium and vitamin D supplementation (500 mg calcium and 200 IU vitamin D) indicated a protective effect of the supplement in peri- and postmenopausal women as those receiving the supplement showed no loss of total BMD, whereas the placebo group did lose BMD [26]. A study by Meier *et al.* [27] looked at the effect of calcium and vitamin D prescribed in winter months. During the first year of the study, calcitropic hormone changes were monitored and then measured the following winter with supplementation. Taking calcium (500 mg) and vitamin D (500 IU) during winter months helped to offset the effects of calcitropic hormones [27]. In another study, adults received vitamin D supplementation (0, 5, 10, 15 µg vitamin D) during wintertime. There was a dosage-related increase of serum vitamin D in the intervention groups and a decrease in PTH [21].

Interventions studying the effect of supplements on falls and fractures focused on vitamin D supplementation. A study by Sanders *et al.* [28] measured the effect of a single, annual dose of 500,000 IU of vitamin D given prior to the start of winter months. The intervention group had a higher number of falls and a relative risk for falls. Kärkkäinen *et al.* [29] conducted a large scale and smaller scale intervention. Both studies measured the effect of 1000 mg calcium and 800 IU vitamin D supplements given to community-dwelling women. In the large scale study, no difference between incidence of single or multiple falls was found but the smaller intervention resulted in a reduction in multiple falls, including those needing medical attention. Elderly, receiving calcium (1000 mg) with vitamin D (800 IU), had a decrease in first falls *versus* those who only received calcium [30]. Elderly, living in residential centers with minimum serum vitamin D level of 25 nmol/L, were given vitamin D (10,000 IU weekly dose and then 1000 IU/day), or placebo [31]. Those receiving the vitamin D supplement had a lower rate of falls and fractures.

### 3.3. Interventions Focusing on Additional Bone-Healthy Nutrients

Research in protein, sodium, soy and vitamin K interventions yielded additional details in bone‑health interventions.

For protein, a cross-sectional intervention by Rapuri *et al.* [32] evaluated the effect of protein as a percentage of energy and its effect of calcium intake. Those in the higher protein quartile (mean 78 grams/day) had higher spine, radius and total BMD *versus* those with lower protein intakes. The effect was seen in those with at least 400 mg calcium.

A crossover study measured the effect of low and high sodium with low and high calcium intake on urinary calcium loss [33]. A diet high in sodium (11.2 g) increased calcium urinary loss leading to negative calcium balance with those on a high calcium diet (mean 1284 mg). Calcium balance was negative for low calcium intake (mean 518 mg), regardless of the sodium intake.

Soy-related interventions on bone-health have had varied results. A calcium and vitamin D supplement with isoflavones showed that the group receiving 120 mg of isoflavones was more protective at the femoral neck BMD [34] and total BMD [35]. Women with low BMD and taking genistein supplements (54 mg) had lower bone resorption markers (pyridinoline and deoxypyridinoline) and increased bone-specific alkaline phosphatase and insulin-like growth factor I. Post-menopausal women consuming 43 grams of soy protein with varying levels of isoflavones resulted in no changes in bone resorptive markers [36].

Varying levels of vitamin K supplementation has been studied. Booth *et al.* [37] measured the effect of an additional 500 μg phylloquinone in older men and women. There was no difference in BMD but the percent of undercarboxylated osteocalcin was reduced in those receiving additional vitamin K. In women with no osteoporosis, groups were supplemented with either a placebo, calcium and vitamin D, vitamin K or calcium (1000 mg), and vitamin D (400 IU) with vitamin K (200 μg) [38]. Those receiving the combination of calcium, vitamin D and vitamin K had an increase in bone mineral content (BMC) and BMD at the radius. Both vitamin K groups had lower percent of undercarboxylated osteocalcin. A study [39] of post-menopausal women measured the effect of a supplement with calcium/magnesium/zinc/vitamin D with or without vitamin K and a placebo. The group taking the combination with vitamin K show decreased bone loss at the femoral neck site [39].

### 3.4. Interventions Using Portfolio Diets

The Dietary Approaches to Stop Hypertension (DASH) and Mediterranean diets have been studied for impact on bone health markers. In an 18-month intervention, participants were randomized to control, standard diet for hypertension and the DASH diet. Those in the DASH diet had significant increases in nutrients positively impacting bone health, including calcium, potassium and magnesium [40]. The effect of the Mediterranean diet on bone markers was studied in elderly men and women [41]. Participants followed either a low fat diet (control), Mediterranean diet with olive oil as the monounsaturated fatty acid (MUFA) source, or Mediterranean diet with nuts as the MUFA source. The group consuming nuts had a higher acid load and slightly higher PTH levels but no other difference in biomarkers.

## 4. Discussion

More than a decade ago the Food and Drug Administration approved the label health claim that calcium-rich products can lower the risk for osteoporosis [42]. Since that time, the relative importance of calcium in bone health and fracture prevention has been an issue of many investigations. A primary function of vitamin D is to maintain serum calcium and phosphorus levels within a constant range, and to enhance calcium availability by intestinal uptake and osteoclast activation. In the last five years, scientific interest in vitamin D has blossomed, both in regards to bone and other medical conditions. Clearly both calcium and vitamin D contribute to healthy bone maintenance during aging, although the relative contribution may still be unclear. Nevertheless, all osteoporosis medication recommends adequate intake of calcium and vitamin D to ensure optimal effectiveness of the drug.

As such, the results of this review are not initially surprising. One finding of this review is that most studies found that vitamin D supplementation would increase serum vitamin D levels [21,22] and an improvement in serum markers of bone health and/or PTH was reported [21,22,23,27]. In addition, BMD was improved with supplementation [23,24,25,26,29] and number of falls were reduced in some [29,30] but not all studies [28]. Our results concerning supplements are similar to other recently published reviews. A meta-analysis of vitamin D alone or vitamin D with calcium supplementation found that vitamin D with calcium was effective in reducing overall fracture risk and hip fracture risk [43]. Indeed this was also reported in a meta-analysis of calcium supplementation on hip fracture. Reid *et al.* reported that hip fracture risk was significantly increased with calcium supplementation alone, but decreased with both calcium and vitamin D supplementation [44]. This is in contrast to the meta‑analysis reported by Tang *et al.* in [45] in which calcium supplementation decreased fracture risk, and little was added to the reduction when vitamin D was added. However a Cochran review concluded that vitamin D alone was ineffective in preventing hip fracture in older people, and although vitamin D with calcium did appear to reduce hip fracture incidence, hypercalcaemia was more common in older persons taking vitamin D with or without calcium, with some increases in gastrointestinal symptoms and renal disease [46]. 

One interesting result of this analysis was that few reviews and no meta-analyses have evaluated food as the source of calcium and/or vitamin D, which are far less likely to produce side effects. Our review found that studies providing calcium as food sources increased calcium and vitamin D intake [15]. When calcium and vitamin D intake was increased, an effect was seen on BMD, bone turnover markers, and PTH [13,14,15]. It is more difficult to quantify calcium or vitamin D intake when these nutrients are eaten as foods rather than as supplements. While there are several validated instruments published for assessing calcium intake in various groups [47,48,49,50,51,52,53,54], very few include calcium-fortified foods [55]. A significant gap in the research includes not only a vitamin D instrument for intake documentation, but a database containing the expanding list of vitamin D-fortified foods.

Of interest is those educational interventions without a food or supplement also increase calcium intake [9,15,19]. Decades of research on nutrition education have found that education would be more effective if focused on specific behaviors and if appropriate theory was used for designing the intervention [56]. A study of older adults (n = 162) found that attitudes and perceived control were important predictors of dairy product intake, whereas subjective norms were not (those significant people who influence our behavior) [57]. Not surprisingly in our review, when the education lacked depth and theory, e.g., mailings to participants, no improvement in calcium intake was found [17]. More importantly, studies examining mediators of bone-related dietary change in older adults are lacking. Similar to demonstrating a physiological mechanism for how nutrients affect health, investigations into educational strategies must investigate how behavioral theories mediate dietary change [58].

Concerning protein, both high and low protein intakes have been shown to have negative effects on bone, with negative effects more pronounced with inadequate calcium intake than with adequate intake [59,60,61]. The study supported a positive effect of protein on bone health. Other studies distinguish between animal and vegetable protein [62], or the acid load of protein and its effects on bone health. A review of all the studies related to bone and protein intake is beyond the scope of this paper. However, an educational interventional approach to adequate calcium, vitamin D, and protein intake with older persons is clearly lacking in the literature. One study did show that protein supplementation offered no additional increase in muscle mass in middle age and older men involved in a resistance exercise randomized control trial [63]. This is mentioned to highlight the other pertinent aspects of bone health, namely muscle mass and exercise.

Other nutritional issues highlighted in our review included vitamin K, soy, and portfolio diets. The latter presents the most holistic approach to investigating the optimal diet for bone health in aging, and may also include exercise and fall prevention training. The studies highlighted in this review demonstrate that key lifestyle habits can be utilized to strengthen bone health and reduce fracture risk. As previously mentioned, the National Osteoporosis Foundation [1] developed five steps to optimize bone health, the first of which is to improve calcium and vitamin D intake. As shown in the present review, this can be accomplished with either food or supplements in older adults. Medications certainly have a role for treating osteoporosis. However, with the number of medications that the older person is prescribed continuing to rise, there is a need to consider less aggressive measures when possible.

While most intervention studies and reviews concerning bone health have included medication as the primary intervention, relatively few have concentrated on diet or supplements alone. Pharmacological approaches will have a significantly higher effect on BMD, and possibly on falls and fractures, but at the same time pose a higher risk for medication-associated side effects, especially if continued for decades.

## 5. Conclusions

Many nutrients play a role in optimizing skeletal mass. In addition to vitamin D and calcium, deficiency, and in some cases excesses, of other nutrients can impact bone mineral density. These nutrients are commonly obtained by following the U.S. Dietary Guidelines [54]. Interventions, including foods or supplements, or targeting behaviors, have shown positive results. Additional research is needed to examine the impact of educational strategies that combine all nutritional factors into one randomized controlled intervention; then further to examine the impact of education plus supplemental foods. The results of these trials will perhaps provide an effective strategy for bone health in aging adults who prefer not to take supplements or medication. Future research may also combine these proposed projects with balance, flexibility, and impact exercise, to determine their additional effects on bone density, fracture rates, and markers of bone health. With the economic factors of supplements and medication, as well as quality of life issues, additional research is needed to examine the potential role of education strategies concerning bone health in aging.

## 6. Limitations

An inherent limitation in evaluating impact of interventions is publication bias. Significant changes are commonly needed in order to be published. If an intervention produced no change, this may not be published and consequently data were not available for review.

With the focus of the review being nutritional interventions, the review excluded studies that focused on drug treatment. These studies may have provided additional nutritional information but the diet variable is often held constant in these studies.

## References

[1] National Osteoporosis Foundation Osteoporosis: A debilitating disease that can be prevented and treated. http://www.nof.org/osteoporosis/index.htm.

[2] Carmona R.H. (2004). Surgeon General. Bone Health and Osteoporosis: A Report from the Surgeon General,. http://www.surgeongeneral.gov/library/bonehealth/.

[3] Compston J. (2010). Osteoporosis: Social and economic impact. Radiol. Clin. North Am..

[4] Pasco J.A., Sanders K.M., Hoekstra F.M., Henry M.J., Nicholson G.C., Kotowicz M.A. (2005). The human cost of fracture. Osteoporos. Int..

[5] Burge R., Dawson-Hughes B., Solomon D.H., Wong J.B., King A., Tosteson A. (2007). Incidence and Economic Burden of Osteoporosis-Related Fractures in the United States 2005–2025. J. Bone Miner. Res..

[6] Al Attia H., Adams B. (2007). Osteoporosis in men: Are we referring enough for DXA and how?. Clin. Rheumatol..

[7] Poole K., Compston J. (2006). Osteoporosis and its management. BMJ.

[8] Gass M., Dawson-Hughes B. (2006). Preventing Osteoporosis-related fractures: An overview. Am. J. Med..

[9] Tussing L., Chapman-Novakofski K. (2005). Osteoporosis prevention education: Behavior theories and calcium intake. J. Am. Diet. Assoc..

[10] Schmitt N.M., Schmitt J., Dören M. (2009). The role of physical activity in the prevention of osteoporosis in postmenopausal women―An update. Maturitas.

[11] Thomas-John M., Codd M.B., Manne S., Watts N.B., Mongey A.B. (2009). Risk Factors for the Development of Osteoporosis and Osteoporotic Fractures Among Older Men. J. Rheumatol..

[12] Shea B., Wells G., Cranney A., Zytaruk N., Robinson V., Griffith L., Hamel C., Ortiz Z., Peterson J., Adachi J. (2004). Calcium supplementation on bone loss in postmenopausal women. Cochrane Database Syst. Rev..

[13] Moschonis G., Katsaroli I., Lyritis G.P., Manios Y. (2010). The effects of a 30-month dietary intervention on bone mineral density: The Postmenopausal Health Study. Br. J. Nutr..

[14] Manios Y., Moschonis G., Katsaroli I., Grammatikaki E., Tanagra S. (2007). Changes in diet quality score, macro- and micronutrients intake following a nutrition education intervention in postmenopausal women. J. Hum. Nutr. Diet..

[15] Bonjour J.P., Benoit V., Pourchaire O., Ferry M., Rousseau B., Souberbielle J.C. (2009). Inhibition of markers of bone resorption by consumption of vitamin D and calcium-fortified soft plain cheese by institutionalised elderly women. Br. J. Nutr..

[16] Hien V.T., Khan N.C., Mai le B., Lam N.T., Phuong T.M., Nhung B.T., Nhien N.V., Nakamori M., Yamamoto S. (2009). Effect of community-based nutrition education intervention on calcium intake and bone mass in postmenopausal Vietnamese women. Public Health Nutr..

[17] Solomon D.H., Finkelstein J.S., Polinski J.M., Arnold M., Licari A., Cabral D., Canning C., Avorn J., Katz J.N. (2006). A randomized controlled trial of mailed osteoporosis education to older adults. Osteoporos. Int..

[18] Sedlak C.A., Doheny M.O., Estok P.J., Zeller R.A. (2005). Tailored interventions to enhance osteoporosis prevention in women. Orthop. Nurs..

[19] Wong S.Y., Lau E.M., Lau W.W., Lynn H.S. (2004). Is dietary counseling effective in increasing dietary calcium, protein and energy intake in patients with osteoporotic fractures? A randomized controlled clinical trial. J. Hum. Nutr. Diet..

[20] Heaney R.P., McCarron D.A., Dawson-Hughes B., Oparil S., Berga S.L., Stern J.S., Barr S.I., Rosen C.J. (1999). Dietary changes favorably affect bone remodeling in older adults. J. Am. Diet. Assoc..

[21] Seamans K.M., Hill T.R., Wallace J.M., Horigan G., Lucey A.J., Barnes M.S., Taylor N., Bonham M.P., Muldowney S., Duffy E.M., Strain J.J., Kiely M., Cashman K.D. (2010). Cholecalciferol supplementation throughout winter does not affect markers of bone turnover in healthy young and elderly adults. J. Nutr..

[22] Kuwabara A., Tsugawa N., Tanaka K., Fujii M., Kawai N., Mukae S., Kato Y., Kojima Y., Takahashi K., Omura K. (2009). Improvement of vitamin D status in Japanese institutionalized elderly by supplementation with 800 IU of vitamin D(3). J. Nutr. Sci. Vitaminol..

[23] Hits M.F., Jensen J.E., Eskildsen P.C. (2007). Bone mineral density and bone markers in patients with a recent low-energy fracture: Effect of 1 y of treatment with calcium and vitamin D. Am. J. Clin. Nutr..

[24] Prince R.L., Devine A., Dhaliwal S.S., Dick I.M. (2006). Effects of calcium supplementation on clinical fracture and bone structure: Results of a 5-year, double-blind, placebo-controlled trial in elderly women. Arch. Intern. Med..

[25] Jackson R.D., LaCroix A.Z., Gass M., Wallace R.B., Robbins J., Lewis C.E., Bassford T., Beresford S.A., Black H.R., Blanchette P. (2006). Calcium plus vitamin D supplementation and the risk of fractures. N. Engl. J. Med..

[26] Di Daniele N., Carbonelli M.G., Candeloro N., Iacopino L., De Lorenzo A., Andreoli A. (2004). Effect of supplementation of calcium and vitamin D on bone mineral density and bone mineral content in peri- and post-menopause women; a double-blind, randomized, controlled trial. Pharmacol. Res..

[27] Meier C., Woitge H.W., Witte K., Lemmer B., Seibel M.J. (2004). Supplementation with oral vitamin D3 and calcium during winter prevents seasonal bone loss: A randomized controlled open‑label prospective trial. J. Bone Miner. Res..

[28] Sanders K.M., Stuart A.L., Williamson E.J., Simpson J.A., Kotowicz M.A., Young D., Nicholson G.C. (2010). Annual high-dose oral vitamin D and falls and fractures in older women: A randomized controlled trial. JAMA.

[29] Kärkkäinen M.K., Tuppurainen M., Salovaara K., Sandini L., Rikkonen T., Sirola J., Honkanen R., Arokoski J., Alhava E., Kröger H. (2010). Does daily vitamin D 800 IU and calcium 1000 mg supplementation decrease the risk of falling in ambulatory women aged 65–71 years? A 3-year randomized population-based trial (OSTPRE-FPS). Maturitas.

[30] Pfeifer M., Begerow B., Minne H.W., Suppan K., Fahrleitner-Pammer A., Dobnig H. (2009). Effects of a long-term vitamin D and calcium supplementation on falls and parameters of muscle function in community-dwelling older individuals. Osteoporos. Int..

[31] Flicker L., MacInnis R.J., Stein M.S., Scherer S.C., Mead K.E., Nowson C.A., Thomas J., Lowndes C., Hopper J.L., Wark J.D. (2005). Should older people in residential care receive vitamin D to prevent falls? Results of a randomized trial. J. Am. Geriatr. Soc..

[32] Rapuri P.B., Gallagher J.C., Haynatzka V. (2003). Protein intake: Effects on bone mineral density and the rate of bone loss in elderly women. Am. J. Clin. Nutr..

[33] Teucher B., Dainty J.R., Spinks C.A., Majsak-Newman G., Berry D.J., Hoogewerff J.A., Foxall R.J., Jakobsen J., Cashman K.D., Flynn A., Fairweather-Tait S.J. (2008). Sodium and bone health: Impact of moderately high and low salt intakes on calcium metabolism in postmenopausal women. J. Bone Miner. Res..

[34] Alekel D.L., Van Loan M.D., Koehler K.J., Hanson L.N., Stewart J.W., Hanson K.B., Kurzer M.S., Peterson C.T. (2010). The soy isoflavones for reducing bone loss (SIRBL) study: A 3-y randomized controlled trial in postmenopausal women. Am. J. Clin. Nutr..

[35] Wong W.W., Lewis R.D., Steinberg F.M., Murray M.J., Cramer M.A., Amato P., Young R.L., Barnes S., Ellis K.J., Shypailo R.J., Fraley J.K., Konzelmann K.L., Fischer J.G., Smith E.O. (2009). Soy isoflavone supplementation and bone mineral density in menopausal women: A 2-y multicenter clinical trial. Am. J. Clin. Nutr..

[36] Cheong J.M., Martin B.R., Jackson G.S., Elmore D., McCabe G.P., Nolan J.R., Barnes S., Peacock M., Weaver C.M. (2007). Soy isoflavones do not affect bone resorption in postmenopausal women: A dose-response study using a novel approach with 41Ca. J. Clin. Endocrinol. Metab..

[37] Booth S.L., Dallal G., Shea M.K., Gundberg C., Peterson J.W., Dawson-Hughes B. (2008). Effect of vitamin K supplementation on bone loss in elderly men and women. J. Clin. Endocrinol. Metab..

[38] Bolton-Smith C., McMurdo M.E., Paterson C.R., Mole P.A., Harvey J.M., Fenton S.T., Prynne C.J., Mishra G.D., Shearer M.J. (2007). Two-year randomized controlled trial of vitamin K1 (phylloquinone) and vitamin D3 plus calcium on the bone health of older women. J. Bone Miner. Res..

[39] Braam L.A., Knapen M.H., Geusens P., Brouns F., Hamulyák K., Gerichhausen M.J., Vermee C. (2003). Vitamin K1 supplementation retards bone loss in postmenopausal women between 50 and 60 years of age. Calcif. Tissue Int..

[40] Lin P.H., Appel L.J., Funk K., Craddick S., Chen C., Elmer P., McBurnie M.A., Champagne C. (2007). The PREMIER intervention helps participants follow the Dietary Approaches to Stop Hypertension dietary pattern and the current Dietary Reference Intakes recommendations. J. Am. Diet. Assoc..

[41] Bulló M., Amigó-Correig P., Márquez-Sandoval F., Babio N., Martínez-González M.A., Estruch R., Basora J., Solà R., Salas-Salvadó J. (2009). Mediterranean diet and high dietary acid load associated with mixed nuts: Effect on bone metabolism in elderly subjects. J. Am. Geriatr. Soc..

[42] FDA (Food and Drug Administration). Title 21: Food and Drugs, Part 101 Food labeling. Subpart E. Specific requirements for health claims. Health claims: Calcium and osteoporosis. http://www.cfsan.fda.gov/~dms/lab-ssa.html.

[43] ART (Vitamin D Individual Patient Analysis of Randomized Trials) Group (2010). Patient level pooled analysis of 68 500 patients from seven major vitamin D fracture trials in US and Europe. BMJ.

[44] Reid I.R., Bolland M.J., Grey A. (2008). Effect of calcium supplementation on hip fractures. Osteoporos. Int..

[45] Tang B.M., Eslick G.D., Nowson C., Smith C., Bensoussan A. (2007). Use of calcium or calcium in combination with vitamin D supplementation to prevent fractures and bone loss in people aged 50 years and older: A meta-analysis. Lancet.

[46] Avenell A., Gillespie W.J., Gillespie L.D., O’Connell D. (2009). Vitamin D and vitamin D analogues for preventing fractures associated with involutional and post-menopausal osteoporosis. Cochrane Database Syst. Rev..

[47] Blalock S.J., Currey S.S., DeVellis R.F., Anderson J.J., Gold D.T., Dooley M.A. (1998). Using a short food frequency questionnaire to estimate dietary calcium consumption: A tool for patient education. Arthritis Care Res..

[48] Blalock S.J., Norton L.L., Patel R.A., Cabral K., Thomas C.L. (2003). Development and assessment of a short instrument for assessing dietary intakes of calcium and vitamin D. J. Am. Pharm. Assoc..

[49] Cook A.J., Friday J.E. (2003). Food mixture or ingredient sources for dietary calcium: Shifts in food group contributions using four grouping protocols. J. Am. Diet. Assoc..

[50] Harnack L.J., Lytle L.A., Story M., Galuska D.A., Schmitz K., Jacobs D.R., Gao S. (2006). Reliability and validity of a brief questionnaire to assess calcium intake of middle-school-aged children. J. Am. Diet. Assoc..

[51] Jensen J.K., Gustafson D., Boushey C.J., Auld G., Bock M.A., Bruhn C.M., Gabel K., Misner S., Novotny R., Peck L., Read M. (2004). Development of a food frequency questionnaire to estimate calcium intake of Asian, Hispanic, and white youth. J. Am. Diet. Assoc..

[52] Montomoli M., Gonnelli S., Giacchi M., Mattei R., Cuda C., Rossi S., Gennari C. (2002). Validation of a food frequency questionnaire for nutritional calcium intake assessment in Italian women. Eur. J. Clin. Nutr..

[53] Ward K.D., Hunt K.M., Berg M.B., Slawson D.A., Vukadinovich C.M., McClanahan B.S., Clemens L.H. (2004). Reliability and validity of a brief questionnaire to assess calcium intake in female collegiate athletes. Int. J. Sport Nutr. Exerc. Metab..

[54] Yanek L.R., Moy T.F., Becker D.M. (2001). Comparison of food frequency and dietary recall methods in African-American women. J. Am. Diet. Assoc..

[55] Plawecki K., Evans E., Mojtahedi M., McAuley E., Chapman-Novakofski K. (2009). Assessing calcium intake in postmenopausal women. Prev. Chronic Dis..

[56] Contento I. (2008). Review of Nutrition Education Research in the *Journal of Nutrition Education and Behavior*, 1998 to 2007. J. Nutr. Educ. Behav..

[57] Kim K., Reicks M., Sjoberg S. (2003). Applying the Theory of Planned Behavior to Predict Dairy Product Consumption by Older Adults. J. Nutr. Educ. Behav..

[58] Cerin E., Barnett A., Baranowski T. (2009). Testing Theories of Dietary Behavior Change in Youth Using the Mediating Variable Model with Intervention Programs. J. Nutr. Educ. Behav..

[59] Dawson-Hughes B. (2003). Interaction of dietary calcium and protein in bone health in humans. J. Nutr..

[60] Kerstetter J.E., O’Brien K.O., Insogna K.L. (2003). Low protein intake: The impact on calcium and bone homeostasis. J. Nutr..

[61] Spence L.A., Weaver C.M. (2003). New perspectives on dietary protein and bone health: Preface. J. Nutr..

[62] Weikert C., Walter D., Hoffmann K., Kroke A., Bergmann M.M., Boeing H. (2005). The relation between dietary protein, calcium and bone health in women: Results from the EPIC-Potsdam cohort. Ann. Nutr. Metab..

[63] Bemben M.G., Witten M.S., Carter J.M., Eliot K.A., Knehans A.W., Bemben D.A. (2010). The effects of supplementation with creatine and protein on muscle strength following a traditional resistance training program in middle-aged and older men. J. Nutr. Health Aging.

